# Identity rupture and reconstruction in post-stroke PTSD: a patient journey map

**DOI:** 10.3389/fpsyt.2026.1796324

**Published:** 2026-06-04

**Authors:** Xiaotong Yu, Li Liu, Hongyang Wu

**Affiliations:** 1 Medical Insurance Department, Binzhou Medical University Hospital, Binzhou, China; 2 School of Nursing, Binzhou Medical University, Binzhou, Shandong, China

**Keywords:** journey map, post- traumatic stress disorder, psychological rehabilitation, qualitative research, stroke, triangulation verification

## Abstract

**Objective:**

To identify the core psychological rehabilitation needs of post-stroke Post-Traumatic Stress Disorder patients based on a patient-family-nurse triad-verified journey map, and to provide references for developing precise nursing intervention strategies.

**Methods:**

A descriptive qualitative research method was adopted. From July 2025 to November 2025, a convenience sample of 19 participants, including post-stroke PTSD patients, primary caregivers, and healthcare professionals from a tertiary general hospital in Binzhou City, were recruited for semi-structured interviews. Content analysis was used to organize the data, and a psychological rehabilitation journey map was constructed with triangulation verification.

**Results:**

A total of 24 sub-themes were summarized in terms of tasks, emotions, and pain points, forming a symptom-experience journey map covering four phases: traumatic impact, support Imbalance, resilience activation, and life stabilization.

**Conclusion:**

This triad-verified journey map visually reveals the phase-specific process of identity reconstruction in post-stroke PTSD and provides a practical framework for developing phased, person-centered interventions.

## 1 Introduction

Stroke is a major global public health issue. Its high mortality and disability rates cause immense suffering for patients, place a heavy emotional and caregiving burden on their families, and increase the economic burden on society (1). Characterized by acute onset and direct threat to life, the suddenness of this disease constitutes an intense traumatic experience (2). In the general population, the lifetime prevalence of Post-Traumatic Stress Disorder (PTSD) ranges from 3.9% to 6.8% (3) and the disorder is associated with significantly reduced quality of life and impaired daily functioning (4). Approximately 20%–33% of stroke survivors subsequently develop PTSD (5). Beyond inflicting psychological distress, PTSD reduces stroke survivors’ rehabilitation compliance and impedes functional recovery, thereby trapping them in a vicious cycle of psychosomatic interaction (6). Effectively identifying the identity reconstruction challenges induced by PTSD among stroke patients across various phases of rehabilitation, and delivering phase-specific interventions, represents the cornerstone of achieving holistic psychosomatic recovery. Although previous studies have confirmed the high incidence and harmfulness of post-stroke PTSD (7), patients’ long-term psychological adaptation processes remain largely undocumented. The retrospective account presented in this study addresses this gap by providing a long-term perspective. The patient journey map is a patient-centered visualization tool that systematically presents patients’ behaviors, emotional changes and service needs during a specific health course in chronological order (8). This method enables not only clinical teams but also patients themselves, families, and community-based support systems to gain deep insights into patients’ psychological and behavioral characteristics at different phases and identify key intervention points. At present, the application of this research method in chronic disease management is gradually increasing (9); however, empirical studies focusing on post-stroke PTSD-specific experiences remain a research gap. Therefore, this study adopts a qualitative research approach, collecting multi-dimensional experiential data from patients, their family members and medical staff through in-depth interviews. It systematically organizes patients’ PTSD-related experiences from the acute phase to the long-term rehabilitation phase, and constructs a symptom experience journey map for post-stroke PTSD patients, which presents the tasks, emotions, and pain points associated with PTSD throughout patients’ transition from the acute phase to long-term rehabilitation. Meanwhile, it explores the mechanism by which identity disruption and reconstruction act on the occurrence, development and rehabilitation of PTSD, providing theoretical and practical evidence for developing phase-specific precision nursing interventions, and ultimately improving the level of whole-course mental health management for stroke patients.

## 2 Subjects and methods

### 2.1 Study subjects

This study adopted a purposive sampling method. From July 2025 to November 2025, post-stroke PTSD patients, their primary caregivers, and medical staff were conveniently selected from a Grade A tertiary general hospital in Binzhou as the main study subjects, so as to ensure that the maximum information could be provided for the research questions. Inclusion Criteria:patients: ① Meeting the diagnostic criteria for stroke as specified in Diagnostic Criteria for Various Major Cerebrovascular Diseases in China (10); ② Disease course ≥ 1 month; ③ Aged ≥ 18 years; ④ Conscious and capable of effective verbal communication; ⑤ Meeting the diagnostic criteria for PTSD as defined in the Diagnostic and Statistical Manual of Mental Disorders (5th Edition) (11); ⑥ Voluntarily participating in this study and signing the informed consent form. Caregivers: ① Serving as the primary caregivers for the above-mentioned patients (with weekly care time ≥ 20 hours); ② Aged ≥ 18 years; ③ Providing informed consent. Nurses: ① Having worked in the Department of Neurology or Department of Rehabilitation for ≥ 3 years; ② Having experience in nursing stroke patients; ③ Providing informed consent. Exclusion criteria: Patients: ① Unable to cooperate with the interview due to complicated other severe physical diseases or mental disorders (e.g., schizophrenia, severe dementia); ② Unable to complete communication after assessment due to severe aphasia or cognitive impairment. This study was approved by the Hospital Ethics Review Committee (Approval No. 2025-KYLL-271). All subjects provided informed consent and participated voluntarily.

### 2.2 Research methods

**Figure 1:**
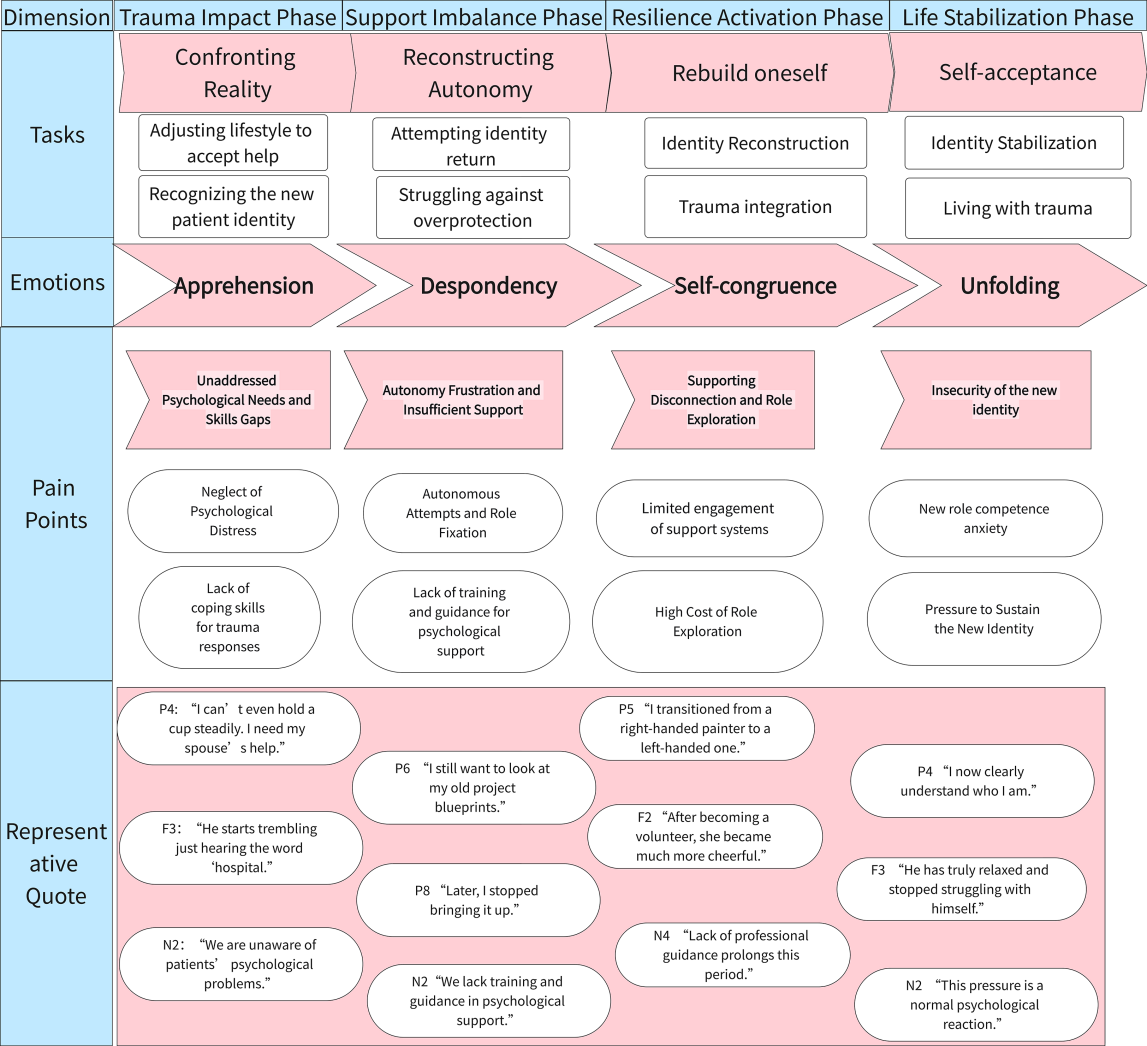
Symptom experience journey map for post-stroke PTSD patients.

The development of the patient journey map aims to systematically illustrate the dynamic changes in patients’ psychological and behavioral experiences along the timeline of the disease course. This study was conducted based on the Consolidated Criteria for Reporting Qualitative Research (COREQ) (12), The research framework was established through literature review and discussions within the research team, which included neurologists, rehabilitation therapists, clinical nursing specialists, and psychology researchers. Data were collected via semi-structured in-depth interviews (13)and analyzed using reflective thematic analysis (14)or data integration and journey map construction (8)。The psychosocial recovery journey of post-stroke PTSD patients was divided into four phases: traumatic impact, support interaction, resilience activation, and life stabilization. The vertical axis of the map, derived from core themes extracted from the interviews, displays the tasks, emotions, and pain points related to PTSD at different phases. Ultimately, a symptom experience journey map for post-stroke PTSD patients was constructed, as shown in **Figure 1**. The corresponding thematic analysis is detailed in **Table 1**.

**Table 1:** Thematic analysis of the post-stroke PTSD journey map.

Phase	Dimension	Theme	Sub-theme	Representative quotes
Trauma Impact Phase	Tasks	Confronting Reality	Adjusting lifestyle to accept help	P4:”The hand that had held a scalpel for a lifetime suddenly wouldn’t obey me. Now I can’t even hold a cup steadily. I need my wife to help me with eating and dressing.”
			Recognizing the new patient identity	P1:”I used to manage more than ten people. Now I can’t even type on a keyboard smoothly. The psychological gap is too huge. I’m still not used to who I am now.”
	Emotions	Apprehension	Fear and helplessness	P2:”Whenever I close my eyes, I see the rescue scene.”
			Anxiety and confusion	P7:”I don’t know what else I can do for this family.”
	Pain Points	Unaddressed Psychological Needs and Skills Gaps	Neglect of Psychological Distress	P4:”No one cares about what is going on in my mind.”
			Lack of coping skills for trauma responses	P2:”I have absolutely no idea how to calm myself down.”
Support Imbalance Phase	Tasks	Reconstructing Autonomy	Attempting identity return	P6:”I used to visit construction sites and negotiate projects. Now I need someone to accompany me just to take a walk downstairs. But I still want to look at my old engineering drawings.”
			Struggling against overprotection	P9:”My family members hand me things and open doors for me. I know they mean well, but they don’t let me do anything on my own. I want to try myself, even if it takes a little longer.”
	Emotions	Despondency	Frustration and disappointment	P6:”I used to understand complicated things at a glance. Now I stare at them for a long time and still can’t figure them out. It makes me feel so frustrated.”
			Self-silencing	P8:”I told my family a few times that I wanted to help my neighbors with something. Every time, they just said, ‘You need to focus on getting better first.’ After a while, I stopped bringing it up.”
	Pain Points	Autonomy Frustration and Insufficient Support	Autonomous Attempts and Role Fixation	P9:”I practice my grip strength every day. But whenever the scene of my stroke flashes through my mind, I feel so panicked that I can’t continue.”
			Lack of training and guidance for psychological support	N2:”Our work priorities and evaluation metrics remain focused on physical rehabilitation, and we ourselves lack training and guidance in psychological support.”
Resilience Activation Phase	Tasks	Rebuild oneself	Identity Reconstruction	P5:”Since I couldn’t draw with my right hand, I practiced with my center. Just being able to hold a paintbrush made me very satisfied. I transitioned from a right-handed painter to a center-handed painter.”
			Trauma integration	P6:”A terrifying image flashed through my mind, so I went for a walk in the park and practiced calligraphy; gradually, my emotions calmed down.”
	Emotions	Self-congruence	Acceptance of the New Identity	P5:”I no longer dwell on the fact that my paintings aren’t as good as they used to be.”
			Sense of Stability	P9:”Gradually, I became able to dress and eat on my own without relying on my family’s assistance. I feel quite competent now.”
	Pain Points	Supporting Disconnection and Role Exploration	Limited Engagement of Support Systems	P6:”My family still asks if I’d like to help, but I tell myself, ’ I’m someone who can manage myself.’”
			High Cost of Role Exploration	P1:”Initially, I listened to music to alleviate anxiety, but the more I listened, the more annoyed I became. Later, I switched to listening to opera, and gradually I calmed down.”
Life Stabilization Phase	Tasks	Self-acceptance	Identity Stabilization	P4:”I now have a clear understanding of who I am—a physician capable of providing health education, and I am very satisfied with this role.”
			Living with trauma	P4:”I live with fear, but I will not be defeated by it.”
	Emotions	Unfolding	Satisfaction and Fulfillment	P4:”I am very satisfied with this situation; I can contribute my value.”
			Composure and Acceptance	F3:”He truly relaxed and stopped struggling with himself. Previously, he had been forcing himself to endure; now, he had genuinely accepted the situation.”
	Pain Points	Insecurity of the new identity	New role competence anxiety	P4:”I’m still worried about having an episode during my lectures, so I always find a place to rest in advance.”
			Pressure to Sustain the New Identity	P3:”If I feel unwell and don’t volunteer for a week, I worry that others might think I’m no longer useful.”

#### 2.2.1 Design of interview outline

Based on the research objectives, a systematic literature search on post-stroke PTSD, psychological resilience, and patient experience was performed to inform the development of the interview guide Following expert review within the research team (15), pilot interviews were carried out with two patients, two family caregivers, and one nurse. The guide was subsequently revised for clarity and appropriateness of questioning based on their feedback, resulting in the final version.

Interview guide for patients: ① Please reflect on your key experiences and feelings from the onset of your condition to the present. ② What have been the most troubling physical and psychological issues for you at different phases since becoming ill, and how have you coped with them? ③ Are there any specific scenes, sounds, or objects that make you feel particularly anxious or afraid? What do you usually do in such situations? ④ How do you feel your role within your family and in society has changed since your illness? How do you perceive these changes? ⑤ What kind of help have you received from your family and healthcare professionals throughout this process? What support has been most valuable to you? ⑥ What are your expectations or plans for the future?

Interview guide for family caregivers: ① Please describe your caregiving experiences and main feelings since your family member became ill. ② What noticeable changes in emotions and behaviors have you observed in them since the onset of the illness? ③ What have been the greatest challenges in providing care, and how have you addressed them? ④ What do you believe they need most in terms of support? In what areas could healthcare professionals or society offer better assistance? ⑤ How has this situation affected your personal life and psychological well-being?

Interview guide for nurses: ① Based on your clinical experience, what common psychological and behavioral reactions have you observed among stroke patients? ② How do you identify potential post-traumatic stress (PTSD) or significant psychological distress in patients? ③ What are the main challenges or obstacles you encounter when providing psychosocial support to patients in clinical practice? ④ In your view, what are the current shortcomings in nursing support for stroke patients regarding their psychological and identity-related needs? ⑤ What suggestions or expectations do you have for better supporting patients and their families?

#### 2.2.2 Data collection and quality control

All researchers involved had received systematic training in qualitative research methodology. Prior to each interview, the researcher thoroughly familiarized themselves with the participant’s background and explained the study’s purpose, significance, voluntary nature, and confidentiality principles in detail, after which informed consent was obtained. Interviews were conducted in a quiet and private room or office. Each interview was completed in a single session, aiming to guide patients and family caregivers in providing a retrospective narrative of their complete post-stroke experience, or to invite nurses to share comprehensive insights based on clinical observations. With consent, all interviews were audio-recorded, and non-verbal cues were documented simultaneously. (e.g., facial expressions, gestures, tone of voice) were documented simultaneously. These notes were used to contextualize the verbatim transcripts during data analysis. For example, when a participant’s verbal response was brief or ambiguous, the accompanying non-verbal cues helped the research team interpret the intended meaning and emotional state. Each session lasted approximately 30–60 minutes. Data collection continued until informational saturation was achieved.

#### 2.2.3 Data analysis methods

Within 24 hours after each interview, the audio recordings were transcribed verbatim into text and anonymized. Content analysis was employed for data analysis, following these specific steps: ① Repeatedly reading the transcripts to gain a comprehensive understanding of the interview content; ② Conducting initial coding of meaningful sentences or paragraphs in the text; ③ Grouping the codes to form preliminary concepts; ④ Further refining the concepts to establish categories and themes; ⑤ Comparing the commonalities and differences among different interviewees to extract core themes; ⑥ Integrating the themes in line with the research objectives to construct a theoretical descriptive framework. Two researchers independently performed coding and theme extraction. In case of any discrepancies in interpretation during the process, discussions were held within the research team until a consensus was reached. Finally, the themes extracted from all interview data were integrated to form the core structure of patient experiences. Based on this structure, a patient journey map was developed to systematically illustrate the changes in patients’ experiences across different phases of the illness.

## 3 Results

### 3.1 General characteristics of participants

A total of 9 patients, 5 primary caregivers, and 5 nurses were finally included in the study. Patients were coded as P1 to P9, primary caregivers as F1 to F5, and nurses as N1 to N5. The general characteristics of the participants are presented in **Table 2**.

**Table 2:** General information of the participants(n=19).

Group classification	Code	Gender	Age (years)	Educational level	Employment status	Residential area	Disease duration/care duration/working years	Treatment method
Patient	P1	Male	52	Senior High School	Retired	Urban	4 months (disease duration)	Thrombolytic Therapy
Patient	P2	Male	48	Junior College	Resigned	Urban	6 months (disease duration)	Interventional Thrombectomy
Patient	P3	Male	55	Senior High School	Unemployed	Rural	12 monts (disease duration)	Conservative Treatment
Patient	P4	Female	43	Master’s Degree	On Leave	Urban	13 months (disease duration)	Thrombolytic Therapy
Patient	P5	Female	67	Bachelor’s Degree	Unemployed	Urban	3 months (disease duration)	Conservative Treatment
Patient	P6	Male	51	Senior High School	Retired	Urban	10 months (disease duration)	Interventional Thrombectomy
Patient	P7	Male	59	Junior High School	Unemployed	Rural	7 months (disease duration)	Conservative Treatment
Patient	P8	Male	62	Primary School	Unemployed	Rural	7 months (disease duration)	Conservative Treatment
Patient	P9	Female	67	Junior High School	Unemployed	Rural	7 months (disease duration)	Interventional Thrombectomy
caregiver	F1	Female	40	Bachelor’s Degree	On Leave	Urban	4 months (care duration for P1)	
caregiver	F2	Male	30	Bachelor’s Degree	On Leave	Urban	3 months (care duration for P4)	
caregiver	F3	Female	49	Technical Secondary School	Resigned	Urban	5 months (care duration for P2)	
caregiver	F4	Male	40	Junior High School	On Leave	Rural	6 months (care duration for P8)	
caregiver	F5	Female	32	Junior College	Resigned	Urban	3 months (care duration for P5)	
Nurse	N1	Female	38	Junior College	Nurse Practitioner	Urban	12 years (working in Department of Neurology)	
Nurse	N2	Female	42	Junior College	Senior Nurse Practitioner	Urban	15 years (working in Department of Rehabilitation)	
Nurse	N3	Female	32	Bachelor’s Degree	Nurse Practitioner	Urban	8 years (working in Department of Neurology)	
Nurse	N4	Female	29	Bachelor’s Degree	Nurse Practitioner	Urban	5 years (working in Department of Rehabilitation)	
Nurse	N5	Female	45	Bachelor’s Degree	Associate Chief Nurse	Urban	18 years (working in Department of Neurology)	

As shown in **Table 2**, the sample demonstrated heterogeneity across sex, age, education, and residence. Among the nine patients, there were six males and three females, aged 43–67 years, with education ranging from primary school to a master’s degree, and five from urban and four from rural areas. The five caregivers included two males and three females, aged 30–49 years. The five nurses were all female, aged 29–45 years, with 5–18 years of experience, including nurse practitioner, senior nurse practitioner, and associate chief nurse.

Member checking was conducted to verify the findings. All five nurses verified the journey map, along with six patients (P1, P3, P4, P5, P6, P9) and three family caregivers (F1, F2, F5). All confirmed that the map accurately reflected their experiences. The remaining three patients and two caregivers could not be contacted after discharge.

From the interviews, a total of 24 sub-themes were identified and organized under three dimensions (tasks, emotions, and pain points). These sub-themes were then distributed across the four phases of the journey map (traumatic impact, support imbalance, resilience activation, and life stabilization), as illustrated in **Figure 1**.

### 3.2 Interview findings

#### 3.2.1 Trauma impact phase

##### 3.2.1.1 Tasks: confronting reality

(1) Adjusting lifestyle to accept help:Patients experience a sudden loss of bodily control, necessitating reliance on others for assistance with fundamental activities of daily living. P4:”The hand that used to hold a scalpel all my life suddenly won’t obey me—I can’t even steady a cup. Now I need my spouse’s help to eat and get dressed.” F1:”My father now requires my full attention for almost all basic daily care. I dare not relax for a moment, worried he might not manage on his own.”N1:”In the early phase, patients have very poor ability to perform daily activities and rely completely on others for care.”.

(2) Recognizing the new patient identity:Patients gradually recognize their sudden change from healthy individuals to patients, a new identity they are not yet ready to accept. P1:”I used to manage over a dozen people. Now I can’t even type smoothly on a keyboard. The psychological gap is huge, and I’m still not used to who I am now.” F4:”Whenever my father mentions his condition or needs help, he can’t help sighing and shows strong resistance.” N5:”Patients often experience confusion about their self-identity—’Who am I now?’—and even resist routine daily care.”.

##### 3.2.1.2 Emotions: apprehension

(1) Fear and helplessness: Flashbacks of traumatic scenes trigger intense fear, while loss of bodily control and forced dependence lead to feelings of helplessness.P2:”As soon as I close my eyes at night, the rescue scene comes back. The doctor’s words—’massive infarction, ’ ‘possible paralysis’—keep ringing in my ears, scaring me so much I can’t sleep.” P9:”I can only follow what my family and doctors say. I never think about what I can do myself—I feel utterly helpless.” F3:”He starts trembling just hearing the word ‘hospital.’ He often wakes up crying from nightmares at night, and I don’t know how to comfort him.”N3:”Early-phase patients are generally sensitive to ‘rescue memories.’ They actively avoid hospital-related cues like white coats or medical settings.”.

(2) Anxiety and confusion: Anxiety arises from uncertainty about the future, along with confusion about self-positioning. P1:”I don’t know what I can do in the future. When I see former colleagues posting about work, I don’t dare comment, afraid they’ll ask what I’m doing now.” P7:”I used to make all the family decisions. Now I need help even to get dressed. I don’t know what I can still contribute to the family—I feel completely lost.”F4:”My father keeps asking, ‘Will I stay like this forever?’ ‘Can I still recover?’ The more he asks, the more anxious he gets—he even loses his appetite.”N5:”Patients’ anxiety isn’t solely due to physical discomfort; they are more worried about permanently losing their sense of worth and purpose.”.

##### 3.2.1.3 Pain points: unaddressed psychological needs and skills gaps

(1) Neglect of psychological distress:Family members and medical staff focus primarily on physical care, with insufficient attention paid to patients’ psychological needs. Generalized reassurance fails to address core distress. P4:”No one understands my fear of becoming useless. My family just says, ‘Don’t overthink it, ’ and the medical staff only ask, ‘How’s your body?’ No one really cares about what’s on my mind.”F3:”I feed him, bathe him, massage him, and tell him, ‘You’ll get better slowly.’ When I see him cry, I assume it’s just physical discomfort—I didn’t think further.”N1:”We mainly focus on vital signs and physical recovery, like whether muscle strength has improved, and rarely discuss psychological states with them.”N2:”We always thought that once the physical issues were treated, the psychological problems would gradually resolve. We are unaware of patients’ psychological problems.”.

(2) Lack of coping skills for trauma responses:Patients lacked the skills to cope with trauma responses, received no guidance, and could only resort to passive avoidance. P2:”Closing my eyes brings back the rescue scene. Hearing an ambulance siren makes me tremble uncontrollably. I can only switch the TV channel or hide in my room to escape—I have no idea how to calm myself down.” P6: “Whenever I see a construction crane, I remember the moment I collapsed. My heart races instantly, and I can only quickly take a detour. I have absolutely no way to stop feeling so afraid.” F2: “Now she gets so nervous at the sight of a white coat or a medical drama that she dares not go out. She won’t even go to the pharmacy near our home unless we accompany her. She feels completely helpless on her own.” N4: “In the early phase, we mistakenly interpreted flashbacks and avoidance as postoperative anxiety, considering them normal. We didn’t recognize these as PTSD triggered by an identity crisis, nor did we teach patients coping methods. As a result, they could only passively avoid, missing the opportunity for early intervention.”.

#### 3.2.2 Support imbalance phase

##### 3.2.2.1 Tasks: reconstructing autonomy

(1) Attempting identity return: After initial stabilization of physical function, patients attempt to return to their former social roles, testing whether they can still fulfill past responsibilities. P6:”I used to be able to run construction sites and negotiate projects. Now I even need someone to accompany me for a walk downstairs. But I still want to look at old project blueprints and see if I can help colleagues organize materials.” P8:”Everyone used to call me ‘Big Brother.’ They’d come to me to mediate conflicts. Now I go out with a cane, but I still feel happy when people greet me. I want to chat with everyone.”F2:”She keeps flipping through her old work notes, saying, ‘I want to go back to work, even if it’s just for half a day.’” N3:”Patients frequently mention their past professional and social relationships, trying to reconnect with their former identity to verify their remaining self-efficacy.”.

(2) Struggling against overprotection: Struggling between dependence on support and the desire for autonomy, patients need basic care but resent overprotection that deprives them of their independence. P9: “My family hands me things and opens doors for me. I know they mean well, but I feel like they treat me as disabled—they don’t let me do anything. I want to try myself, even if it’s slower.” F1: “I’m afraid my father might fall, so I try to help him get dressed. But he gets angry and says, ‘I can do it myself; you don’t need to manage me.’ We’ve argued about this several times.”N2: “Family members often proactively help patients with tasks they could manage themselves, like handing them a water cup or tying their shoelaces, unintentionally depriving them of autonomy. Patients often secretly complain to us that they want to do things themselves.

##### 3.2.2.2 Emotions: despondency

(1) Frustration and disappointment: Wanting to return to their former roles but being unable to do so. P6:”I used to grasp a complex situation at a glance. Now I can’t quite understand even after looking for a long time. It’s very frustrating.” P8:”I want to help neighbors mediate disputes, but now I speak slowly. What’s in my mind doesn’t come out of my mouth smoothly. I feel very disappointed in myself.” F2: “She wants to return to work, but her current physical capacity can no longer meet the demands of her previous job.” N4:”Patients often tell us, ‘It’s disappointing to think I can’t do my old job. I feel like I’ve worked hard all my life, and suddenly my professional ability is gone.’”.

(2) Self-silencing: Patients develop emotional distance and barriers from others. They become unwilling to share their true feelings and thoughts, actively closing the door to communication. P8:”I mentioned a few times to my family that I’d like to help the neighbors with something. Each time they said, ‘Just focus on taking care of yourself.’ Later, whenever I had such thoughts, I kept them to myself and didn’t bring them up again.” F2:”Later I noticed she never mentioned work to me again. Even when I ask, she doesn’t talk about it.” N4:”Patients often tell us, ‘No one understands my desire to be useful. There’s no point in talking about it.’”.

##### 3.2.2.3 Pain points: autonomy frustration and insufficient support

(1) Autonomous attempts and role fixation: patients want to take control of their lives or rehabilitation, but their PTSD symptoms dominate them. P9: “I practice my grip in front of the mirror every day and slowly try to dress and eat by myself. But if a scene from the attack flashes through my mind, I get so panicked that I have to stop practicing and rest for a while.” F3: “He was willing to walk around the convenience store near home—finally a step forward. But as soon as he passes the road near the hospital, even from a distance, his face suddenly turns pale and he dares not go further. His previous courage vanishes instantly.” N3: “Patients have shifted from ‘passive endurance’ to ‘active attempts.’ However, this sense of autonomy is still fragile. When confronted with entrenched traumatic responses, their autonomous attempts are easily interrupted and difficult to sustain, trapping them in a dilemma where they desire autonomy but remain confined to the patient role”.

(2) Lack of training and guidance for psychological support: neither healthcare providers nor family caregivers know how to support patients’ psychological needs. N2:”Our work focus and evaluation metrics are still centered on physical rehabilitation progress. We ourselves lack the training and guidance on how to truly connect with patients psychologically.” F1:”Seeing him start to practice on his own makes us very happy, but we don’t know what else we can do. We just watch from the side, afraid he might fall, or hand him a cup of water when he’s tired. It feels like what we can do is still the same as before.”.

#### 3.2.3 Resilience activation phase

##### 3.2.3.1 Tasks: rebuild oneself

(1) Identity reconstruction: rebuilding identity helps patients restore a sense of self-satisfaction and self-worth. P5: “Since I can no longer paint with my right hand, I practice with my left. Although the result is not as good as before, I feel satisfied just being able to hold a brush—shifting from a right-handed painter to a left-handed one.” P3: “Now I am a rehabilitation volunteer, helping newly diagnosed patients ease their anxiety. I never thought my own illness could be of help to others—transitioning from a patient to a volunteer.” F2: “After becoming a volunteer, she became much more cheerful and said she now feels useful.” N5: “Patients now identify sources of value compatible with their current condition by adapting their former roles or establishing new ones.”.

(2) Trauma integration: the patient begins to accept the traumatic reactions as part of themselves, actively explores ways to coexist with the trauma, and shifts from passive suffering to active regulation. P4: “I still feel nervous when I see a hospital, but that’s all right. I accept that I have these symptoms. As long as they don’t interfere with my health education work.”P6: “If a frightening scene flashes through my mind, I go for a walk in the park, practice Tai Chi, or stay at home reading or practicing calligraphy. These activities help my emotions gradually calm down.”P1: “If I feel panicky or anxious, I take deep breaths or listen to traditional opera, and that slowly helps me settle.”F3: “During that period, he was particularly reluctant to go out, especially when passing near the hospital. Later, he began practicing dressing on his own. Although he was slow, he became more willing to move than before, and developed more ways to cope with difficulties on his own.”N3: “Patients’ initiative in self-regulation has strengthened, which proves more effective than medical intervention.”.

##### 3.2.3.2 Emotions: self-congruence

(1) Acceptance of the new identity: Patients have come to accept that their former identity cannot be restored. P5: “I’m satisfied just being able to hold a brush. I no longer dwell on the fact that my paintings aren’t as good as they used to be.”P8: “I teach chess at the community senior center. Feeling needed again gives me a strong sense of reassurance and purpose.” F5: “She has now accepted painting with her left hand, saying, ‘It’s different, but it’s still my painting.’” N5: “Patients’ attitude toward the illness has shifted from resistance to acceptance, and they have developed a sense of identification with their new roles.”.

(2) Sense of stability: The patient follows a routine life. Witnessing his own gradual improvement, he experiences a sense of inner reassurance and stability. P9: “Gradually, I can dress and eat by myself. Although it’s slow, I no longer have to wait for family assistance. I feel I can still manage.”P1: “I planned my own rehabilitation schedule and stuck to it for three months. My hand strength has improved a lot, and I feel I can regain control over my life.”P6: “Being able to handle daily life without relying on family gives me a great sense of accomplishment.”F1: “Now he plans his own rehab and even shares every little progress with us—he appears very confident.”N2: “By completing rehabilitation independently and realizing new value, patients gradually rebuild their self-confidence.”.

##### 3.2.3.3 Pain points:supporting disconnection and role exploration

(1) Limited engagement of support systems: support systems remain confined to instrumental care, unable to understand or engage in identity reconstruction and symptom management. Patients must rely entirely on self-exploration, lacking external emotional resonance. P6: “Family still asks if I need help, but I tell myself ‘I am someone who can manage myself, ’ and handle what I can on my own.” F1: “He now sees himself as a ‘rehabilitation coach, ’ plans his own training. We can only help with groceries. What he specifically thinks or plans, we do not really know.” F2: “She plans everything she wants to do herself, like volunteering in the community. I just need to support her.” N2: “Patients’ active self-regulation is more effective than medical intervention, yet we healthcare providers still do not actively participate in patients’ psychological adjustment process.”.

(2) High cost of role exploration: costs: With no universal approach to symptom relief and no guidance, each patient must identify what works for them through trial and error, a process that cannot be bypassed. P1: “At first, I tried listening to music to relieve anxiety, but it only made me more upset. Later, I switched to opera and gradually calmed down.” P6: “Initially, I used ‘deep breathing’ to cope with flashbacks, but it made me more tense. Later, I tried ‘walking’ and found exercise more useful.” F3: “He tried several methods—sometimes they worked, sometimes they didn’t. We couldn’t help, just watched him figure it out on his own.” N4: “It is necessary for the patient to autonomously explore ways to achieve self-stabilization, but the current lack of professional guidance prolongs this period and increases psychological pressure.”.

#### 3.2.4 Life stabilization phase

##### 3.2.4.1 Tasks: self-acceptance

(1) Identity stabilization: patients develop a clear and stable self-perception, no longer needing external validation. P4: “I now clearly understand who I am—I am a doctor who can conduct health education and help young doctors review medical records. I am very satisfied with this state.”P3: “I volunteer at the hospital every week for rehabilitation support, sharing my experiences with newly diagnosed patients. Helping them gives me a sense of purpose, and I hope to maintain this lifestyle.”F3: “He now has a very clear sense of what he wants to do. His daily schedule is structured and regular, unlike before when he felt lost.”N5: “The patient’s self-identity has formed a complete cycle, no longer requiring external validation—this is a sign of identity stability.”.

(2) Living with trauma: Patients have integrated PTSD symptoms as a normal part of life, without letting the condition disrupt their daily functioning. P5: “I still occasionally dream about the onset of the illness and wake up sweating, but I’m used to it now. After waking, I look at the paintings I’ve made or think about the people I’ve helped, and it doesn’t seem so frightening anymore.”P4: “I’m still afraid of having an episode in public, so I try to avoid crowded places. But that doesn’t stop me from doing health education in the community. I live with fear, but I won’t be defeated by it.”F3: “He still has nightmares occasionally, but he can quickly adjust on his own and carry on with his day—it doesn’t affect his normal life at all.”N1: “The patient has established a lifestyle of ‘coexisting with trauma, ’ where the presence of symptoms is no longer a primary source of distress.”.

##### 3.2.4.2 Emotions: unfolding

(1) Satisfaction and fulfillment: patients feel satisfied, and their lives feel full and meaningful.P4: “I feel very content with this state—I am able to contribute my value.”P8: “When life is full and meaningful, I don’t dwell on those frightening thoughts.”F1: “He is cheerful every day now and says his life feels purposeful.”N5: “The patient feels satisfied with their new identity and meaningful activities, deriving a sense of fulfillment through ongoing value creation.”.

(2) Composure and acceptance: patients no longer struggle against themselves and have truly accepted their illness experience. P8: “Helping with housework and sharing recovery experiences with neighbors—this grounded feeling is good.”F3: “He has truly relaxed now, no longer struggling inwardly. Before, he was just enduring; now, he genuinely accepts things and even comforts us not to worry.”N4: “The patient has fully accepted the illness experience as part of themselves. They no longer reject themselves due to PTSD symptoms, nor do they demand changes in external support.”.

##### 3.2.4.3 Pain points: insecurity of the new identity

(1) New role competence anxiety: even after making significant progress, patients still harbor concerns about symptom recurrence.P4: “Even though I can do community education, I still worry about having an episode during a session, so I always find a resting spot in advance.”F3: “He can adjust quickly after a nightmare, but I can sense there’s still some underlying worry in him.”N2: “This pervasive pressure concerning the ability to sustain the new identity is not pathological anxiety. Instead, it constitutes a normal psychological reaction within the context of ‘living with trauma.’”.

(2) Pressure to sustain the new identity: patients’identification with their new identity is fragile, accompanied by a persistent anxiety about potentially losing it.P3: “If I feel unwell and miss a week of volunteering, I start wondering if they’ll think I’m no longer useful.”P8: “If I’m not well enough to teach chess someday, I might feel useless again.”F2: “She values her volunteer identity deeply. If she misses a session, she comes back somewhat subdued.”N4: “Patients must invest considerable ongoing effort to maintain their new identity. If physical fluctuations due to relapse risk prevent participation in activities, they may temporarily experience a crisis of lost self-worth.”.

## 4 Discussion

### 4.1 Early nursing strategies in the impact phase

Stroke, as a sudden traumatic event, can induce PTSD. This study found that during the acute trauma impact phase, some patients had already developed PTSD symptoms such as flashbacks and avoidance. Patients have not yet completed the psychological transition from healthy individual to patient role, and struggle to accept their dependence on care resulting from physical function loss. Consistent with Hall et al.’s (16) concept of liminality, these patients described being caught in an uncertain state between their “past healthy self” and “present patient self.” This identity rupture and role adaptation disorder manifest as resistance to the sick role, questioning of self-worth, fear of the present situation, and anxiety about the future. More importantly, patients require psychological empathy for their identity change, whereas families and the healthcare system prioritize instrumental care and medicalized reassurance. This finding highlights the urgent need for psychological support. Hall et al. (16) A similarly found that post-stroke psychological support is frequently insufficient, with only 10.2% of patients receiving it. Meanwhile, when confronted with trauma responses such as flashbacks and avoidance, patients lack effective coping strategies and are unable to calm themselves, resorting to passive escape. Therefore, care at this phase should not be limited to physical care but should simultaneously address psychosocial needs. Based on our findings, the following three strategies may provide a useful framework to guide nursing practice, although further validation is required: ① Simultaneous assessment of trauma symptoms and identity adaptation difficulties: In the early phase of admission, standardized tools such as the PCL-5 (17) to assess the severity of PTSD symptoms. Simultaneously, clinical observation and interviews should be conducted to proactively identify early signs of identity adaptation difficulties.② Targeted communication: For patients, an “empathic communication” technique should be used, in which healthcare providers employ phrases such as” ‘From … to…’ this role transition must be extremely difficult “to accurately acknowledge the patient’s identity discrepancy and emotional experience (18). For patients, this approach accurately acknowledges the patient’s identity discrepancy and emotional experience, replacing generalized reassurance with deep empathy. For family members, it should be clarified that “identity rupture” is a core trigger of PTSD, guiding them to shift from instrumental care to incorporating psychological support. However, this study found that family caregivers already bear a significant caregiving burden and generally lack the skills necessary for providing psychological support. Therefore, while guiding families to provide psychological support, it is necessary to simultaneously offer corresponding training and psychological debriefing services for family caregivers to alleviate their caregiving burden. ③Provision of identity stabilization tools: To help patients initially stabilize themselves when PTSD-related symptoms emerge, Simple psychological stabilization techniques, such as “sensory grounding” (19) and “safe place imagery” (20), should be taught promptly. Family members should also be trained to assist with these techniques, thereby establishing an early coping barrier. The implementation of the above strategies relies on the active engagement of formal care structures. Nevertheless, the current medical rehabilitation system has significant deficiencies in providing psychosocial support. Therefore, it is urgently necessary to integrate such support protocols into routine nursing workflows, rather than relying on patients and their families to navigate these challenges on their own.

### 4.2 Optimizing support models to disrupt the “attempt–frustration” cycle of self-efficacy

As physical function initially stabilizes, patients enter a critical transition period in which they test their self-efficacy. Patients began to actively review their work notes and attempt to return to their previous social roles. This aligns with Albert Bandura’s self-efficacy theory (21), which posits that individuals rebuild agency through familiar actions after a loss of ability. However, a dual contradiction characterizes this phase: first, while patients’ psychological needs shift toward autonomous attempts, the reality of over-protective family care and a medical focus on physical rehabilitation creates a tension between the desire for autonomy and persistent dependency, often accompanied by repeated frustration due to role misrecognition and ineffective action; second, patients’ nascent autonomy remains fragile, and the absence of a supportive system easily fosters loneliness, while sudden PTSD symptoms can directly interrupt the rehabilitation process. Therefore, care in this phase must both transform the lagging support model and reinforce fragile autonomy. On one hand, families should be guided to shift from taking over care tasks to appropriately stepping back, encouraging patients to complete manageable activities independently—such as dressing or tidying personal items. On the other hand, healthcare providers should incorporate patients’ willingness to attempt autonomous rehabilitation behaviors into routine rehabilitation assessments and use this as a basis for formulating individualized rehabilitation plans (22).Concurrently, a “symptom-interruption intervention” task may be considered: during rehabilitation training, a pre-set intervention procedure is activated when PTSD symptoms disrupt the practice. This triggers a standardized”3-minute rapid-calming” protocol, helping the patient stabilize emotionally and immediately return to the task, thereby preserving behavioral continuity (23).

### 4.3 Self-management intervention pathway for driving cognitive restructuring and identity compensation

When the expectation of restoring their former identity fades, patients enter a breakthrough phase in psychological rehabilitation. In this phase, patients complete cognitive and emotional reconstruction, shifting from resisting illness to accepting their current situation and anchoring themselves in new roles—such as regaining a sense of value and control through identities like left-handed painter or rehabilitation volunteer. This process is consistent with the findings of Hall et al. (24) in their digital storytelling study with stroke survivors: engagement in new role practices helped patients regain a sense of value and control. During this phase, patients transition from passive symptom bearing to active self-management, forming an “acceptance → empowerment → consolidation” cycle. However, challenges remain: First, support engagement is limited—family and medical staff remain confined to basic care, struggling to empathize with patients’ new-role needs; second, trial-and-error costs are high—despite willingness, patients lack professional knowledge and are prone to repeated failures when seeking individualized symptom management methods, which depletes their psychological energy and confidence. To address these issues, it is necessary to guide family members to understand the value of patients’ new roles, shifting from a task-centered care model to emotional companionship and participatory support, while fully recognizing the complexity and emotional burden inherent in all caregiving work. Healthcare providers should offer personalized symptom management guidance: based on the patient’s symptom profile, psychiatric-mental health nurses should develop tailored coping plans. During the active symptom management period, the focus of care shifts to providing evidence-based, individualized symptom response strategies. For example, for patients experiencing flashbacks, an intervention integrating cognitive techniques and mindfulness practices may be designed (25); for patients with a tendency toward avoidance, a laddered task approach to support behavioral activation may be planned (26) to reduce blind trial and error.

### 4.4 Long-term care atrategies for maintaining dynamic balance in “Coexisting with Trauma”

The ultimate goal of the rehabilitation journey for post-stroke PTSD patients is not the complete elimination of PTSD symptoms, but the establishment of a dynamic balance in which patients can coexist with their symptoms without being dominated by them. However, this balance is dynamic and requires active maintenance. Two key challenges emerge at this phase: first, the concern about symptom recurrence, where even during stable periods, patients experience underlying anxiety about symptom relapse in critical situations; second, the fragility of the new identity, as patients’ sense of positive value and emotional stability heavily depend on the consistent practice of their new roles, and any interruption in these activities may easily lead to emotional lows marked by a loss of self-worth. During this phase, healthcare providers can help patients develop symptom warning and response plans. Healthcare providers can guide patients to document symptom-triggering scenarios, such as public spaces or medical settings, and proactively plan coping strategies. A diverse value support system may be considered to guide patients in expanding sources of self-worth beyond a single role. For example, extending from the identity of “rehabilitation volunteer” to include roles such as “family experience sharer” and “community health promoter” helps prevent over-reliance on any one role for validation. During follow-up, healthcare professionals may consider affirming patients’ intrinsic qualities and inherent value, rather than focusing solely on the external outcomes of role performance (27).

## 5 Conclusion

Based on the perspectives of patients, family members, and nurses, this study constructed a four-phase symptom experience journey map of post-stroke PTSD, visually presenting patients’ psychosocial adaptation needs across different rehabilitation phases—from “traumatic impact” to “coexisting with trauma”—through three dimensions: tasks, emotions, and pain points. The study found that the core tension in post-stroke PTSD lies in identity disruption and reconstruction, with patients encountering challenges such as support-need misalignment, restricted autonomy, and role rigidity at different phases. These findings suggest that healthcare providers should attend to patients’ identity adaptation difficulties at each rehabilitation phase, establish an identity-reconstruction and phase-matched psychosocial support system, and develop individualized symptom warning and response plans.

## 6 Limitations

First, the retrospective design may introduce recall bias, as patients with PTSD may have difficulty accurately recalling past experiences or may unconsciously reconstruct their memories. Second, the sample was limited in terms of clinical characteristics; this study did not systematically collect data on stroke severity, lesion location, or aphasia status. Third, the study was conducted in a single geographical region. Although thematic saturation was achieved, the findings may not fully represent the rehabilitation experiences of post-stroke PTSD patients from different regions. Future research should adopt prospective designs, systematically collect clinical characteristic data, expand clinical and demographic diversity, and conduct multicenter longitudinal studies to better capture patients’ psychosocial support needs across different rehabilitation phases.

## Data Availability

The raw data supporting the conclusions of this article will be made available by the authors, without undue reservation.

## References

[1] Tu WJ Zhao Z Yin P Cao L Zeng J Chen H . Estimated burden of stroke in China in 2020. JAMA Netw Open. (2023) 6:e231455. doi: 10.1001/jamanetworkopen.2023.1455. PMID: 36862407 PMC9982699

[2] Cameron TM Walker MF Fisher RJ . A qualitative study exploring the lives and caring practices of young carers of stroke survivors. Ijerph. (2022) 19:3941. doi: 10.3390/ijerph19073941. PMID: 35409626 PMC8997658

[3] Koenen KC Ratanatharathorn A Ng L McLaughlin KA Bromet EJ Stein DJ . Posttraumatic stress disorder in the World Mental Health Surveys. Psychol Med. (2017) 47:2260–74. doi: 10.1017/S0033291717000708. PMID: 28385165 PMC6034513

[4] Jellestad L Vital NA Malamud J Taeymans J Mueller-Pfeiffer C . Functional impairment in posttraumatic stress disorder: A systematic review and meta-analysis. J Psychiatr Res. (2021) 136:14–22. doi: 10.1016/j.jpsychires.2021.01.039. PMID: 33548826

[5] Tang WK Wang L Tsoi KKF Rutovic S Kim JS . Post-traumatic stress disorder after stroke: A systematic review. Neurol India. (2022) 70:1887–95. doi: 10.4103/0028-3886.359285. PMID: 36352583

[6] Faizal M Zulkifly M Ezzat S . The ability of recovery locus of control scale (RLOC) and post-traumatic stress symptoms (PTSS) to predict the physical functioning of stroke patients. 22:31–41. PMC5295747 28239266

[7] Nie YP Bai HM Zhao G Jiang C . Research progress on post-stroke post-traumatic stress disorder [Research progress on post-stroke post-traumatic stress disorder]. Chin J Clin Neurosurg [Chinese Journal of Clinical Neurosurgery]. (2022) 27:407–11. doi: 10.13798/j.issn.1009-153X.2022.05.025. in Chinese with English abstract.

[8] Bulto LN Davies E Kelly J Hendriks JM . Patient journey mapping: Emerging methods for understanding and improving patient experiences of health systems and services. Eur J Cardiovasc Nurs. (2024) 23:429–33. doi: 10.1093/eurjcn/zvae012. PMID: 38306596

[9] Dai MQ Liao XQ . Advances in patient journey mapping in the care of patients with chronic diseases [Advances in patient journey mapping in the care of patients with chronic diseases]. J Nurs Sci [Journal of Nursing Science. (2024) 39:121–5. doi: 10.3870/j.issn.1001-4152.2024.13.121. in Chinese with English abstract.

[10] Chinese Society of Neurology Chinese Stroke Society . Diagnostic criteria of cerebrovascular diseases in China (version 2019) [Diagnostic criteria of cerebrovascular diseases in China (version 2019)]. Chin J Neurol [Chinese Journal of Neurology]. (2019) 52:710–5. doi: 10.3760/cma.j.issn.1006-7876.2019.09.003. PMID: . in Chinese with English abstract. 30704229

[11] Sharp C Miller JD . Head-to-head comparisons of diagnostic and statistical manual of mental disorders, fifth edition, section II and section III personality disorder in predicting clinical outcomes. Pers Disorders: Theory Research Treat. (2024) 15:275–81. doi: 10.1037/per0000691. PMID: 39235913

[12] Yang L Yang ZY Ruan H . Introduction to the standards for reporting qualitative research (SRQR) and enlightenment for qualitative research development [Introduction to the standards for reporting qualitative research (SRQR) and enlightenment for qualitative research development]. J Nurs Sci [Journal of Nursing Science]. (2019) 34:105–8. doi: 10.3870/j.issn.1001-4152.2019.14.105. in Chinese with English abstract.

[13] Kallio H Pietilä A Johnson M Kangasniemi M . Systematic methodological review: Developing a framework for a qualitative semi‐structured interview guide. J Advanced Nurs. (2016) 72:2954–65. doi: 10.1111/jan.13031. PMID: 27221824

[14] Braun V Clarke V . Supporting best practice in reflexive thematic analysis reporting in Palliative Medicine: A review of published research and introduction to the Reflexive Thematic Analysis Reporting Guidelines (RTARG). Palliat Med. (2024) 38:608–16. doi: 10.1177/02692163241234800. PMID: 38469804 PMC11157981

[15] Polit DF Beck CT . The content validity index: Are you sure you know what’s being reported? Critique and recommendations. Res Nurs Health. (2006) 29:489–97. doi: 10.1002/nur.20147. PMID: 16977646

[16] Hall J Van Wijck F Kroll T Bassil-Morozow H . Stroke and liminality: Narratives of reconfiguring identity after stroke and their implications for person-centred stroke care. Front Rehabil Sci. (2024) 5:1477414. doi: 10.3389/fresc.2024.1477414. PMID: 39691857 PMC11651291

[17] Forkus SR Raudales AM Rafiuddin HS Weiss NH Messman BA Contractor AA . The posttraumatic stress disorder (PTSD) checklist for DSM–5: A systematic review of existing psychometric evidence. Clin Psychology: Sci Pract. (2023) 30:110–21. doi: 10.1037/cps0000111. PMID: 37378352 PMC10292741

[18] Barker M-E Leach KT Levett-Jones T . Patient’s views of empathic and compassionate healthcare interactions: A scoping review. Nurse Educ Today. (2023) 131:105957. doi: 10.1016/j.nedt.2023.105957. PMID: 37734368

[19] Invitto S Moselli P . Exploring embodied and bioenergetic approaches in trauma therapy: Observing somatic experience and olfactory memory. Brain Sci. (2024) 14:385. doi: 10.3390/brainsci14040385. PMID: 38672034 PMC11048503

[20] Raz S Lahad M . Physiological indicators of emotional arousal related to ANS activity in response to associative cards for psychotherapeutic PTSD treatment. Front Psychiatry. (2022) 13:933692. doi: 10.3389/fpsyt.2022.933692. PMID: 36419970 PMC9676269

[21] Murphy JW Shotwell-Tabke C Smith DL Valdespino-Hayden Z Patton E Pridgen S . Evaluating self-efficacy as a treatment mechanism during an intensive treatment program for posttraumatic stress disorder. psychol Trauma: Theory Research Practice Policy. (2025) 17:1192–201. doi: 10.1037/tra0001836. PMID: 39818951 PMC13138502

[22] Blake HT Davis A Mellow ML Hull M Robins B Laver K . Co-design of a digital 24-hour time-use intervention with older adults and allied health professionals. Front Digit Health. (2025) 7:1544489. doi: 10.3389/fdgth.2025.1544489. PMID: 40487986 PMC12141298

[23] Niles B Lang A Olff M . Complementary and integrative interventions for PTSD. Eur J Psychotraumatol. (2023) 14:2247888. doi: 10.1080/20008066.2023.2247888. PMID: 37655624 PMC10478588

[24] Hall J Kroll T Van Wijck F Bassil-Morozow H . Co-creating digital stories with UK-based stroke survivors with the aim of synthesizing collective lessons from individual experiences of interacting with healthcare professionals. Front Rehabilit Sci. (2022) 3:877442. doi: 10.3389/fresc.2022.877442. PMID: 36189023 PMC9397888

[25] Boyd JE Lanius RA McKinnon MC . Mindfulness-based treatments for posttraumatic stress disorder: A review of the treatment literature and neurobiological evidence. J Psychiatry Neurosci. (2018) 43:7–25. doi: 10.1503/jpn.170021. PMID: 29252162 PMC5747539

[26] Wagner AW Jakupcak M Kowalski HM Bittinger JN Golshan S . Behavioral activation as a treatment for posttraumatic stress disorder among returning veterans: A randomized trial. Ps. (2019) 70:867–73. doi: 10.1176/appi.ps.201800572. PMID: 31337325

[27] Calhoun CD Stone KJ Cobb AR Patterson MW Danielson CK Bendezú JJ . The role of social support in coping with psychological trauma: An integrated biopsychosocial model for posttraumatic stress recovery. Psychiatr Q. (2022) 93:949–70. doi: 10.1007/s11126-022-10003-w. PMID: 36199000 PMC9534006

